# SOCIAL NETWORK INFLUENCES ON SENSE OF CONTROL AND ATTRIBUTED DIGNITY IN OLDER AGE: RESEARCH RESULTS

**DOI:** 10.1093/geroni/igz038.3247

**Published:** 2019-11-08

**Authors:** Raeann G LeBlanc, Cynthia Jacelon

**Affiliations:** University of Massachusetts Amherst, Amherst, Massachusetts, United States

## Abstract

The purpose of this study was to explore the relationship between the functions of individual social networks, defined here as social support, and the outcomes of sense of control and attributed dignity among a sample of older people living with multiple chronic conditions. This study integrated an explanatory sequential (Quan/Qual) mixed methods design. Descriptive statistics were used to describe social networks. Bivariate correlations and regression statistics were used to examine the relationships of social network support (MOS-Social Support Scale) with the dependent variables of sense of control (Wallhagen Revised PCQ Questionnaire) and attributed dignity (Jacelon Attributed Dignity Scale). Open-ended interviews and thematic analysis were used to expand understanding of the quantitative findings. A cross-sectional sample of eighty-nine community dwelling older people living with multiple chronic health conditions participated. Social support, as a function of one’s social network, predicted the outcome of sense of control (β = .33, p ≤ .01) and attributed dignity (β = .44, p ≤ .001). Correlation statistics and regression models substantiated positive relationships of social supports’ influence on perceived sense of control and attributed dignity. Thematic analysis, based on open-ended interviews (n=12), expanded on the nuances of social influences on sense of control and attributed dignity in managing chronic health conditions through the themes “learning to ask for help”, “only a phone call away” and “smaller circles”. This research proposes new ways of understanding the relationships between perceptions of social support, sense of control and attributed dignity in later life in managing health.

